# Thermal Tolerance in Anuran Embryos with Different Reproductive Modes: Relationship to Altitude

**DOI:** 10.1155/2013/183212

**Published:** 2013-05-23

**Authors:** Manuel Hernando Bernal, John D. Lynch

**Affiliations:** ^1^Grupo de Herpetología, Eco-Fisiología & Etología, Departamento de Biología, Universidad del Tolima, Calle 42 Barrio Santa Helena, Ibagué, Colombia; ^2^Instituto de Ciencias Naturales, Universidad Nacional de Colombia, Carrera 45 No. 26-85, Bogotá, Colombia

## Abstract

Anurans are ectothermic animals very sensitive to temperature, mainly during the embryonic stage. In addition, environmental temperature decreases with altitude, and the amphibian fauna changes. Therefore, we studied the relationship between the embryonic thermal tolerances of twelve species of anurans and the temperatures of their microhabitat along an altitudinal gradient from 430 m to 2600 m. We hypothesized that there is a strong thermal adjustment of embryos to their microhabitat and, consequently, that temperature could be a limiting factor of altitudinal distribution of the anurans. We also compared the embryonic thermal tolerances according to six postulated reproductive modes of the study species. We found a significant relationship between the maximum and minimum thermal tolerances of the anuran embryos and the maximum and minimum temperatures of their microhabitat and altitudinal distribution. We also found a wide range of embryonic thermal tolerances for aquatic breeding species and a narrower range for terrestrial breeding species. Particularly, embryos of direct development species were the most sensitive to temperature. These results show the strong thermal adjustment of anuran embryos to their microhabitat and elevation and do not reject the hypothesis that temperature can be a limiting factor of their altitudinal distribution.

## 1. Introduction

The distribution of a species, in a broad geographic sense as well as in a narrow ecological sense, is a result from the interaction of multiple intrinsic and extrinsic factors to the individual organisms [1]. However, according to the species, one or a few of these factors may dominate and become critical as a limiting factor of the distribution. Anurans are ectothermic animals very sensitive to different environmental factors, but temperature is one of the most important [2–4]. As ectotherms, anurans do not have a physiological mechanism for thermoregulation, and their corporal temperature is strongly affected by the environmental temperature [5–8]. Thusly, environmental temperature could limit the extension of geographic distribution of these species to habitats where they can maintain appropriate temperatures for survival. It can be still more significant for anuran embryos, which are more sensitive to temperature than adults [1, 9, 10].

Previous works have demonstrated that the thermal tolerances of anuran embryos are correlated with their geographic distribution and breeding habitats [1, 9, 11, 12], although not latitudinally [13]. However, there are no data about this relation along altitudinal gradients in anurans. Janzen [14] argued that thermal gradients in a mountain represent a barrier to dispersal of ectothermic organisms. Also, Lynch [15] and Navas [3] suggested that, given the thermal clines along tropical altitudinal gradients, temperature should be the most important abiotic factor limiting the anuran's distribution and diversity in the Andean mountains. Therefore, we set out to determine the embryonic thermal tolerances of twelve species of anurans to establish if there is a significant relationship to the temperature of their microhabitats and altitudinal distributions. Additionally, we report the thermal tolerances of anuran embryos according to six reproductive modes. Only Townsend and Stewart [16] mentioned that aquatic embryos of the temperate zone are less sensitive to changes of temperature than are terrestrial embryos of *E. coqui*.

## 2. Materials and Methods

### 2.1. Study Species

 We studied embryos of twelve anuran species with six postulated reproductive modes, distributed in Colombia from 430 m to 2600 m elevation (Table 1). Embryos of eight species were collected from the field in an early stage of development, *Rhinella humboldti* (Gallardo 1965) (from five clutches)*, R. marina* (Linnaeus 1758) (four clutches)*, Hypsiboas crepitans* (Wied 1824) (three clutches)*, Dendropsophus microcephalus *(Cope 1886) (five clutches)*, Engystomops pustulosus *(Cope 1864) (four clutches)*, Leptodactylus insularum *(Barbour 1906) (one clutch)*, Espadarana prosoblepon *(Boettger 1892) (seven clutches), and *Sachatamia punctulata* (Ruiz-Carranza & Lynch 1995) (four clutches), and transported to the University of Tolima, Ibagué, Colombia, where the experiments were carried out. In the other four study species, *Dendropsophus labialis *(Peters 1863) (from two clutches)*, Dendrobates truncatus* (Cope 1861) (24 clutches)*, Pristimantis uranobates *(Lynch 1991) (one clutch), and *Eleutherodactylus johnstonei* (Barbour 1914) (13 clutches), embryos were obtained naturally in terrariums at similar temperatures to their habitats. Numbers of embryos for each experimental treatment are shown in the figures.

### 2.2. Experimental Procedure

 Anuran embryos in stage 10 [17] for aquatic breeding species and stage 2-3 [18] for direct development species were randomly assigned to relatively constant experimental temperatures so as to reach stages 25 and 15, respectively, which was the end-point of the experiments. Water baths and refrigerators were used to get the experimental temperatures in an air-conditioned room in the laboratory. Temperatures could not be maintained precisely, and the range of variation was about 1°C. Aquatic embryos were placed in small plastic dishes of 40 mL of capacity, in the proportion of 10 embryos per 20 mL of previously aerated water, whereas two or three terrestrial embryos were placed on wet towels in small petri dishes (51 mm). After that, the plastic dishes and the petri dishes were positioned in plastic containers of 14 cm of length, 10 cm of width, and 4 cm of depth into the water baths and refrigerators. Sensors of digital thermometers were placed in each plastic and petri dish so as to check the experimental temperatures constantly. 

A daily renewal of the embryos' water was made with warm water (35°C), environmental water (25°C), and cold water (15°C), for experiments at high (higher than 30°C), medium (between 20°C and 30°C), and low temperatures (lower than 20°C), respectively. At this time, embryos were examined, and it was carefully noted the presence of dead embryos or any abnormalities in development, such as exogastrulations, imperfect, or retarded development. At 25°C, the dissolved oxygen of water was 6.67 mg/L, pH = 8.33, conductivity = 302 *μ*S/cm, hardness = 90 mg/L CaCO_3_, and alkalinity = 78 mg/L CaCO_3_. At 15°C, the dissolved oxygen was 7.0 mg/L, and, at 35°C, it was 6.18 mg/L. The other physical-chemistry parameters were approximately similar among all different temperatures.

### 2.3. Environmental Temperatures

Temperatures of the embryos' microhabitat were obtained with a Lascar USB data logger (Contoocook, NH, USA) for the terrestrial data, and with a MicroLite 16L data logger (Contoocook, NH, USA) for the aquatic data. The data loggers were placed in the field, where egg clutches were frequently found, and they were programmed to register temperatures each hour from one to two months. Environmental data are reported as maximum, minimum, and mean of the microhabitat temperatures.

### 2.4. Data Analysis

 Curves of survival percent in relation to experimental temperatures were constructed to establish the range and limits of embryonic thermal tolerances. The relationship between the maximum and minimum thermal tolerances and the maximum and minimum microhabitat temperatures, respectively, was analyzed by the Spearman correlation test [19]. For comparison among species, a thermal limit was taken at which more than 70% of the embryos reached successfully the end-point of the experiments (see above), as a more restrictive parameter than the commonly used 50% of survival of embryos [1, 9, 20].

## 3. Results

**Figure 1:**
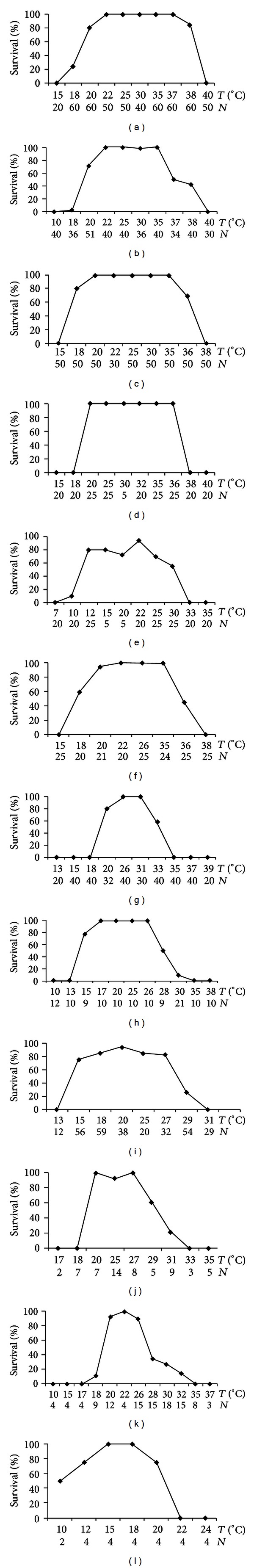
Thermal tolerances in anuran embryos. N: number of embryos per each experimental temperature (°C). (a) *R. humboldti;* (b) *R. marina;* (c) *H. crepitans;* (d) *D. microcephalus;* (e) *D. labialis*; (f) *E. pustulosus*; (g) *L. insularum*; (h) *E. prosoblepon*; (i) *S. punctulata*; (j) *D. truncatus*; (k) *E. johnstonei*; (l) *P. uranobates*.

Anuran embryos for lowland species, collected between 430 and 630 m altitudes, such as *H. crepitans, D. microcephalus, R. humboldti, *and* R. marina,* had a survival higher than 70% at temperatures between 20°C and 36°C (Figures 1(a), 1(b), 1(c), and 1(d)), but temperatures lower than 20°C reduced the survivorship of embryos to almost 0%, with the exception of *H. crepitans* at 18°C, which was 80% (Figure 1(c)). On the other hand, temperatures higher than 37°C decreased the survivorship of embryos by more than 50%, with the exception of *R. humboldti*, where the survival at 38°C was 79% (Figure 1(a)). For these species, the mean microhabitat temperature was between 23°C and 35°C (Table 2). In the highland Andean species *D. labialis,* the range of experimental temperatures with embryonic survival higher than 70% was between 12°C and 25°C, and the mean microhabitat temperature was between 12.3°C and 17.8°C (Table 2).

In the foam nest species, *E. pustulosus,* there was a survival higher than 90% between 20°C and 35°C, but temperatures out of this range decreased the survivorship of embryos below 60% (Figure 1(f)). The mean of the maximum and minimum environmental temperatures for this species was between 22°C and 34°C (Table 2). For *L. insularum*, there was a survival higher than 70% between 20°C and 32°C (Figure 1(g)), and the mean range of the microhabitat temperatures was between 24°C and 28°C (Table 2). In *E. prosoblepon* and *S. punctulata*, collected at 1150 m altitude, there was a survivorship of embryos higher than 70% at temperatures between 15°C and 27°C (Figures 1(h) and 1(i)), and the maximum and minimum microhabitat temperatures were close to these thermal limits (Table 2). With respect to *D. truncatus*, between the minimum (22°C) and maximum (29.5°C) microhabitat temperatures (Table 2), embryos were able to survive at more than 60% (Figure 1(j)); however, at temperatures out of the range, the survivorship decreased strongly.

Finally, in the two direct development species, *E. johnstonei* and *P. uranobates*, the narrowest range of embryonic thermal tolerances was found. In *E. johnstonei*, there was a survival higher than 90% at temperatures between 20°C and 26°C, but temperatures out of this range caused high embryonic mortality (Figure 1(k)). Meanwhile, *P. uranobates* showed a survival less than 75% at temperatures below 12°C or above 20°C (Figure 1(l)), which are close to the limits of their environmental temperatures (Table 2). To summarize these results, nine of the twelve study species had a survival between 90% and 100% at their mean microhabitat temperatures, and the other three species had a survival higher than 70%.

**Figure 2:**
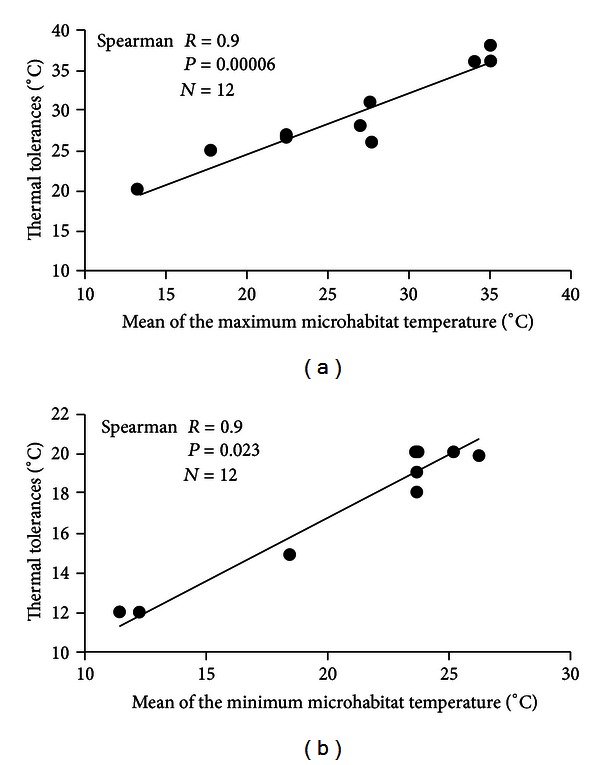
Relationship between the mean of the embryonic microhabitat temperatures and the maximum (a) and minimum (b) embryonic thermal tolerances.

**Figure 3:**
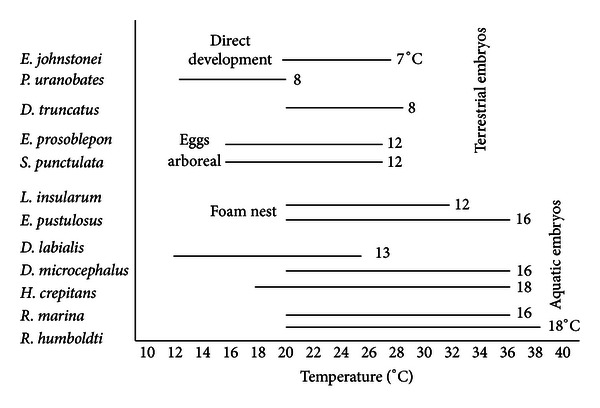
Range of thermal tolerances of the study species for an embryonic survival higher than 70%.

A significant positive relationship between the mean of the maximum microhabitat temperatures and the maximum embryonic thermal tolerances for a survival higher than 70% of the species was found (Spearman *R* = 0.90, *P* = 0.00006, and *N* = 12) (Figure 2(a)). Also, for the relationship between the mean of the minimum microhabitat temperatures and the minimum embryonic thermal tolerances (Spearman *R* = 0.90, *P* = 0.023, and *N* = 12) (Figure 2(b)). On the other hand, comparing the embryonic thermal tolerances according to the reproductive modes of the species for a survival higher than 70%, terrestrial embryos had a range between 7°C and 8°C; arboreal and foam nests embryos were between 12°C and 16°C and aquatic embryos between 13°C and 18°C (Figure 3). Thus, terrestrial embryos had a narrower range than aquatic embryos, and direct development species had the lowest range.

## 4. Discussion

### 4.1. Embryonic Thermal Tolerances in relation to Altitude

There is a significant positive correlation between microhabitat temperatures and embryonic thermal limits (Figure 2). But also three main groups can be detected, one of them with the highest microhabitat temperatures and thermal tolerances, which is integrated by lowland species, collected from 430 m to 827 m altitude, another with intermediate microhabitat temperatures and embryonic thermal tolerances, with species collected at 1200 m altitude, and finally a third group with the lowest microhabitat temperatures and thermal tolerances, which has the two highland species collected at 2600 m altitude. Thus, a decrease of the environmental temperature along an altitudinal gradient is in concordance with a decrease in the embryonic thermal tolerance. These results, therefore, show the thermal adjustment or thermal adaptation *sensu lato* between the embryonic thermal tolerances and the altitudinal distribution of the anuran species. 

Previous studies have found a correlation between the thermal tolerances of anuran embryos and their geographic distribution [1, 21–24], but there are not reports along altitudinal gradients. Other works have found this altitudinal relationship in adults, however, with the methodology of the critical thermal maximum (CT_Max_) and critical thermal minimum (CT_Min_) proposed by Hutchison [25], Heatwole et al. [26], and Lutterschmidt and Hutchison [27]. For instance, Christian et al. [28] reported that *E. portoricensis* located at 700 m altitude had a lower CT_Min_ than *E. coqui* found at 15 m altitude. Also, Spellerberg [29], and Huang et al. [30], working on lizards from Australia and Taiwan, respectively, established that species from high altitudes had a lower critical temperatures than species from lowland places. Other studies have found this inverse relationship between the CT_Max_ and altitude for amphibian populations from temperate zones [5, 31–33] but not for tropical species of Puerto Rico, such as tadpoles of *Leptodactylus albilabris* [34] and adults of *Eleutherodactylus coqui* [28].

Thermal adjustments of anuran embryos can be also detected in relation to different microhabitat temperatures at the same altitude. For instance, *D. truncatus* is a lowland species with terrestrial embryos which develop in a sheltered microhabitat with little variation of the environmental temperature (Table 2) and has a narrower thermal tolerance range than embryos of other species of the same locality (*H. crepitans* and *D. microcephalus*, Figures 1(c) and 1(d)), which develop on the surface of waters exposed to intense sunny days and cold nights. Another example is registered in the two sympatric study species with foam nests. Embryos of *E. pustulosus* develop in foam nest floating on water in open areas and have a wider microhabitat temperatures and thermal tolerances than embryos of *L. insularum *(Figures 1(f) and 1(g)), which develop in shaded places [35]. 

### 4.2. Embryonic Thermal Tolerances in relation to Reproductive Modes

Comparison of embryonic thermal tolerances indicates that species of reproductive modes 1 and 2 have the highest range of thermal tolerances, species of reproductive modes 3 and 4 have an intermediate range of tolerances, and species of reproductive modes 5 and 6 have the lowest range (Figure 3). Therefore, it could be generalized that (1) aquatic anuran embryos, which develop in lowlands and open areas, have a wider range of thermal tolerances than terrestrial embryos and (2) embryos of direct development species have the lowest range of thermal tolerances and consequently are the most sensitive to temperature. Zweifel's work [1] also demonstrated a wide range of thermal tolerances for eight anuran species with aquatic reproduction of the arid southwest of the United States; these ranges were a little more than 17°C for *Spea hammondii* and 19.5°C for *Lithobates pipiens* and *Spea bombifrons*. In another study, Townsend and Stewart [16] reported a temperature range for a normal embryonic development of *Eleutherodactylus coqui* from 20.5°C to 25.0°C and a Q_10_ of 3.92, which was higher than that for any temperate frog species of aquatic breeding, except for*Ascaphus truei*. According to these data, Townsend and Stewart [16] suggested that aquatic embryos are less sensitive to changes in temperature than are terrestrial embryos, as was found in the present study.

Direct development species, as those of the genus *Pristimantis* and *Eleutherodactylus*, can be found in Colombia from the sea level to more than 4000 m altitude [36, 37]. Species with aquatic reproduction and larval development can be also distributed from the sea level, as *Rhinella marina*, to high mountains as the Andean frog *D. labialis* found at 4000 m altitude. Additionally, species with an intermediate reproductive mode, as arboreal embryos with aquatic tadpoles, can be also found in lowlands, for example, *Cochranella ramirezi* at 60 m [37], and highlands, for example, *Centrolene buckleyi* at 3450 m altitude [38]. Thus, it appears that there is not a physiological restriction of the reproductive modes for the altitudinal distribution of their species, although highlands in Colombia are mainly dominated by direct development species of the genus *Pristimantis* [15, 37].

Aspects related to the reproductive modes, such as the color and size of the eggs and clutches, do not show a clear relationship to the thermal sensitivity of embryos. For example, wide ranges of embryonic thermal tolerances are found in species with white eggs deposited in foam nests on ponds (*E. pustulosus*), brown and black eggs deposited as a film on lentic water (*D. microcephalus *and *H. crepitans*, resp.), and black egg strings deposited in lotic or lentic water (*R. marina*). On the other hand, a high thermal sensitivity is registered in the white eggs of the direct development species (*E. johnstonei* and *P. uranobates*) and the terrestrial black eggs of *D. truncatus*. The eggs of the last three species were the most sensible to temperature and also the largest. This suggests a positive relationship between the anuran egg size and the embryonic thermal sensitivity, but it is a hypothesis to test.

## 5. Conclusions

This study found a strong correlation between the thermal tolerances of anuran embryos and the temperatures of their microhabitat. Thus, it can represent the thermal adjustment of the species to their microhabitat and altitude and does not reject the hypothesis that temperature can be a limiting factor of the altitudinal distribution in anurans. Also, aquatic embryos had a wider thermal tolerance than do terrestrial embryos, and embryos of the direct development species were the most sensible to temperature.

## Figures and Tables

**Table 1:** Geographic localization and reproductive modes of the study species in Colombia. Categorizations of the reproductive modes, *sensu* Duellman and Trueb [2], are indicated in parentheses on the right side.

Species	Place	Altitude	Reproductive mode
*R. marina* and *R. humboldti *	Payandé (4°19′N; 75°06′W)	630 m	(1) String eggs deposited in lotic water (1)
*H. crepitans* and *D. microcephalus *	Potrerillo (4°14′N; 74°58′W)	430 m	(2) Superficial film of eggs deposited in lentic water (2)
*D. labialis *	Bogotá (4°38′N; 74°04′W)	2600 m	(2) Egg mass deposited in lentic water (2)
*E. pustulosus* and *L. insularum *	Ibagué (4°21′N; 75°06′W)	827 m	(3) Foam nest deposited on ponds (8)
*S. punctulata *and *E. prosoblepon *	Falan (5°07′N; 74°58′W)	1150 m	(4) Arboreal eggs. Tadpoles drop into streams (18)
*D. truncatus *	Potrerillo (4°14′N; 74°58′W)	430 m	(5) Terrestrial eggs. Tadpoles carried to water by adult (14)
*P. uranobates *	El Silencio (4°36′N; 75°20′W)	2600 m	(6) Terrestrial eggs with direct development (17)
*E. johnstonei *	Bucaramanga (7°07′N; 73°07′W)	950 m	(6) Terrestrial eggs with direct development (17)

**Table 2:** Daily microhabitat temperatures for the study species.

Altitude and place	Species	Embryos microhabitat	*T*_*Max*⁡_ mean (sd)	*T*_*Min*⁡_ mean (sd)	*T*_Range_
(1) *40 m (Barranquilla)	*E. johnstonei *	Land and shaded place	27.73 (0.93)	26.25 (0.53)	25.5–31 (*N* = 575)
(2) 430 m (Potrerillo)	*H. crepitans *and *D. microcephalus *	Water and exposed to sun	34.03 (3.38)	23.71 (1.03)	21.5–43.5 (*N* = 836)
	*D. truncatus *	Land and shaded place	27.03 (1)	25.23 (1.43)	22–29.5 (*N* = 374)
(3) 630 m (Payandé)	*R. marina* and *R. humboldti *	Water and exposed to sun	35.04 (3.53)	23.62 (0.68)	22.5–40.5 (*N* = 306)
(4) 827 m (Ibagué)	*E. pustulosus *	Foam nest on pond exposed to sun	34.0 (4.3)	21.8 (1.12)	19–40.5 (*N* = 309)
	*L. insularum *	Foam nest on pond in a shaded place	27.63 (1.64)	23.88 (1.77)	20.5–31.5 (*N* = 276)
(5) 1150 m (Falan)	*S. punctulata *and* E. prosoblepon *	On leaves in a shaded place	22.43 (1.6)	18.47 (1)	16.5–28.5 (*N* = 862)
(6) 1200 m (Ibagué)	*E. johnstonei *	Land and shaded place	24.16 (3.84)	22.83 (0.35)	22.0–26.0 (*N* = 225)
(7) 2600 m (Ibagué)	*P. uranobates *	Land and shaded place	17.01 (1.06)	12.24 (1.77)	10.1–21.2 (*N* = 1005)
(8) 2600 m (Bogotá)	*D. labialis *	Water (10 cm below the surface)	17.8 (1.91)	12.3 (0.98)	11–20.5 (*N* = 330)

SD: standard deviation; *N*: number of data; *T*_*Max*⁡_: maximum temperature (°C); *T*_*Min*⁡_: minimum temperature; *T*_Range_: range of temperature. *Data obtained for information of microhabitat temperatures where this species is frequently found at the sea level in Colombia.
